# Roles and expression profiles of long non‐coding RNAs in triple‐negative breast cancers

**DOI:** 10.1111/jcmm.13327

**Published:** 2017-09-22

**Authors:** Xiangyi Kong, Wenyue Liu, Yanguo Kong

**Affiliations:** ^1^ Department of Neurosurgery Peking Union Medical College Hospital Chinese Academy of Medical Sciences Beijing China; ^2^ Department of Breast Oncology National Cancer Center/Cancer Hospital Chinese Academy of Medical Sciences and Peking Union Medical College Beijing China; ^3^ Plastic Surgery Hospital Chinese Academy of Medical Sciences and Peking Union Medical College Beijing China; ^4^ Tissue Engineering and Wound Healing Laboratory Department of Surgery Division of Plastic Surgery Brigham and Women‘s Hospital and Harvard Medical School Boston MA USA

**Keywords:** Long non‐coding RNAs, triple‐negative breast cancer, expression

## Abstract

Triple‐negative breast cancer (TNBC) refers to the breast cancers that express little human epidermal growth factor receptor 2 (HER2), progesterone receptor (PR) and oestrogen receptor (ER). When compared to other types of breast cancers, TNBC behaves more aggressively with relatively poorer prognosis. Moreover, except chemotherapy, no targeted treatments have been approved yet until now. Although the molecular‐biological mechanisms of the initiation and development of TNBC have been explored a lot, the exact details underlying its progressions are still not clear. Long non‐coding RNAs (lncRNAs), with the length greater than 200 nucleotides, are non‐protein coding transcripts. Previous researches have shown that lncRNAs are significantly involved in a variety of pathophysiological processes such as cell migration, invasion, proliferation, differentiation and development. lncRNAs’ dysregulated expressions have been observed in many types of tumours including TNBCs. This article will review the functional roles and dysregulations of lncRNAs in TNBCs. These lncRNAs are worthy of exploitation regarding their potential application values of TNBC's diagnosis and treatment.

## Introduction

TNBCs account for almost 20% of all types of breast cancers across the world (approximately, 0.2 million cases per year). TNBCs tend to be more usually diagnosed in young females (<40‐year‐old) than hormone‐positive breast cancers. According to Trivers *et al*.'s 1 survey data, there were twofold higher attributable risks of TNBCs in ≤40‐year‐old females than >50‐year‐old females. Additionally, TNBCs are more common among black women than white women. Histopathologically, TNBCs are more likely to be of high grade (mostly are infiltrating ductal carcinomas, though a rare histologic subtypes, medullary carcinoma, is also generally triple‐negative) 2. TNBC can exhibit geographic necroses, stromal lymphocytic responses and pushing borders of invasion 3. By definition, TNBCs lack immunohistochemical expressions of HER2, PR and ER. As the three biomarkers are currently the only known approved therapeutic targets of breast cancer, considerable effort has been made to better understand other biological forces driving TNBC 3, 4.

Although the TNBCs mostly consist of the basal‐like molecular subtype, considerable heterogeneities within TNBCs still exist. As examples, in one study of utilizing DNA and RNA profiling of TNBCs, four stable subtypes were identified: basal‐like immune‐activated, basal‐like immunosuppressed, mesenchymal and luminal androgen receptor 5. Other molecular‐biological mechanisms underlying TNBC's development include mutations and dysregulated expressions of many DNA repair genes [*e.g*. breast cancer susceptibility gene (BRCA)] 6 and tumour‐suppression genes such as p53 7. These molecular features may have implications for chemotherapy sensitivity to platinum or other directly DNA‐damaging agents. The identification of effective biomarkers for early diagnosis of TNBCs and a better understanding of the systems of the neoplastic advancement are consequently keenly awaited.

## LncRNA and cancers

LncRNAs, with the length greater than 200 nucleotides, are non‐protein coding transcripts. Their roles in cancer initiation and progression have been explored a lot 8. Although LncRNAs were initially considered to be only a promiscuous RNA polymerase‐II activity or a transcriptional noise, research increasingly suggests that lncRNAs play important roles in a repertoire of biological processes, including cell invasion, apoptosis, proliferation, differentiation and development 9. Various mechanisms have been observed in biological experiments, including microRNA sequestration, mRNA stability regulation, pre‐mRNA splicing, RNA polymerase‐II negative‐regulation, recruitment of co‐activators, chromatin remodelling and so on. As an example, lncRNA HOTAIR was found to be overexpressed in patients with breast cancer and could repress the expressions of metastasis‐suppression genes, thus aggravating cancer metastases 10. Another example is lncRNA SNHG12, a cell‐cycle regulator, which is down‐regulated in breast cancer tissues 11. LncRNA CCAT2, UCA1 and H19 have all been reported in a number of neoplasms referring with metastases, apoptosis, survival and proliferation 12. Hence, lncRNA regulates gene expressions and exert influences on molecular‐biological functions through various mechanisms.

An increasing number of researches have demonstrated the dysregulated expressions of lncRNAs in varieties of cancers, including breast cancer, cervical cancer, prostate cancer, oesophageal squamous cell carcinoma, colorectal cancer, nasopharyngeal carcinoma, hepatocellular carcinoma, osteosarcoma and gastric cancer 13. One of the commonest dysregulation forms of lncRNA is elucidated by Iyer *et al*.'s 14integrative analyses of approximately 7200 RNA‐sequencing libraries from cancer and non‐cancer tissues and cells, which identified over 8000 cancer‐related lncRNAs. These deregulated lncRNAs could function as tumour‐suppressor genes or oncogenes. Overexpressions of such onco‐lncRNAs or silencing or repression of anti‐oncogenes could aggravate the malignancy, including cell reprogrammed energy metabolism, apoptosis resistance, over‐angiogenesis, metastasis, increased invasiveness, replicative senescence, growth‐suppression resistance and sustained proliferations. Together, these studies demonstrate that lncRNAs play critical roles in various biological and pathological processes, including apoptosis, cell proliferations and tumourigenesis.

## Profiles of lncRNA expressions in TNBCs

Augoff *et al*.^15^ in 2012 first published the study on lncRNA expression profiles in TNBC. In their report, they identified that the hypermethylation of gene promoter is an important mechanism to silence miR‐31 in basal‐subtype TNBC cells, then mapped miR‐31 to the intronic sequences of a new lncRNA LOC554202, which could regulate the transcriptional activity of miR‐31 15. Both the lncRNA LOC554202 and miR‐31 are up‐regulated in luminal‐subtype cells and down‐regulated in basal‐subtype TNBC cells. Additionally, through using techniques of bisulphite‐converted DNA sequencing and methylation‐specific PCR, they showed that the LOC554202 promoter‐associated CpG island is significantly hypomethylated in the luminal‐subtype cells and significantly methylated in the basal‐subtype TNBC cells 15.

Further literatures related to the profiles of lncRNA expressions in TNBC also detected a series of dysregulated lncRNAs. By virtue of transcriptome microarrays on 165 TNBC samples, Liu *et al*.^16^ made a detailed study on the transcriptome profiling. By calculating the empirical cumulative distribution functions using k‐means clustering, they could work very well on determining optimal numbers of TNBC subtypes. These TNBC samples could be divided into four subtypes: basal‐like and immune suppressed (BLIS) subtype, mesenchymal‐like subtype (MES) subtype, luminal androgen receptor subtype (LAR) subtype and immunomodulatory (IM) subtype. One of the most up‐regulated lncRNAs in the IM‐subtype TNBC was ENST00000443397. In the LAR‐subtype TNBC, expressions of lncRNA ENST00000447908 were increased. In the MES‐subtype TNBC, the most up‐regulated lncRNA was NR_003221. In the BLIS‐subtype TNBC, the most up‐regulated lncRNA was TCONS_00000027 16. Besides lncRNAs expression microarray analysis, more and more bioinformatics methods have been used for lncRNA exploration. Recently, Koduru *et al*.^17^ analysed online available small RNA‐sequencing database derived from 24 TNBC samples and 14 adjacent non‐cancer tissue samples and re‐mapped various subtypes of non‐coding RNAs. They found 61 lncRNAs, among which, 33 were down‐regulated (top 5: lnc‐ZNF75D‐2:2, lnc‐FLOT2‐1:1, lnc‐NEK8‐2:1, lnc‐FLT3LG‐1:7 and lnc‐PAPLN‐2:1) and 28 were up‐regulated (top 5: lnc‐ELP4‐3:1, lnc‐EIF2C2‐1:1, lnc‐PURA‐2:1, lnc‐SC5DL‐3:1 and lnc‐DNAJC16‐1:1) 17.

## Long intergenic non‐coding RNA for kinase activation (LINK‐A)

LINK‐A, also called NR_015407 or LOC339535, is a ∼1.5 kb intergenic lncRNAs 18. LINK‐A is considered to play a significant role in the growth‐factor‐mediated HIF1α cell signal transduction pathway. According to Lin *et al*.'s study, LINK‐A expression levels were much higher in 2 stage‐III TNBC tissues than in their paired adjacent non‐cancer breast tissues, ERPR+/HER2+, HER2‐/ERPR+ and ERPR−/HER2+ breast cancer tissues, suggesting the close association between TNBC and LINK‐A expressions 18. Consistently, they also found that overexpressed LINK‐A in TNBC tissues and cells are associated with poorer prognoses and progression‐free survivals 18. Moreover, different from typical nuclear lncRNAs, they found that LINK‐As are mainly located adjacent to cellular membranes or in cytoplasm 18. LINK‐A could promote tumourigenesis in TNBC through LINK‐A‐dependent signalling pathway activation. Hence, targeting LINK‐A, with much promising therapeutic potential, might be able to provide a favourable strategy to block the HIF1α signalling pathway in TNBCs. Whether LINK‐As are released into circulation continuously through cell apoptosis or actively secreted from TNBC cells through exosome pathways still needs further research.

## The HOX transcript antisense intergenic lncRNA (HOTAIR)

HOTAIR is a 2.3 kb non‐coding transcript derived from the intergenic region of the HOXC homeotic gene cluster 12. HOTAIR plays a critical role in oncogenesis, whose expressions significantly increased in a variety of cancers including hepatocellular carcinoma 19, gastric cancer 20, intestinal cancer 21 and breast cancer 22. HOTAIR overexpression correlates with more severe tumour distant metastases and poorer prognosis. HOTAIR was the first lncRNA shown to promote tumour progression and be related to poor prognosis in breast cancer 23. HOTAIR was involved in regulating malignant biological behaviour of TNBC through a variety of ways. HOTAIR expression was significantly up‐regulated by oestrogen in TNBC cells MDA‐MB‐231 and BT549 and increased the migration of them. HOTAIR can also indirectly suppress certain miRNA expressions in TNBC, reverse epithelial‐mesenchymal transition (EMT) partially, decrease the breast cancer stem cell population, and attenuate cell metastasis and invasion 24. Considering the key roles of HOTAIR in TNBC, attenuating HOTAIR functions to gain treatment effects is promising 12. Yang *et al*. also found Delphinidin‐3‐glucoside could down‐regulate HOTAIR expressions in TNBC cells in both *vitro* and *vivo*. These studies elucidate some unidentified mechanism in TNBC linking signalling with HOTAIR regulation which may be exploited for therapeutic gain 25. These researches identified several mechanisms underlying TNBC's genesis and progression. HOTAIR dysregulation is the most important mechanism, which offers a new target and orientation for TNBC therapy.

## Rhabdomyosarcoma 2‐associated transcript (RMST)

RMST, located on chromosome 12q21 in human beings, was initially reported in rhabdomyosarcomas and was found to be expressed at lower levels in embryonal rhabdomyosarcomas than in alveolar rhabdomyosarcomas 26. Uhde *et al*. found that RMST exhibits prominent expressions in regions of the roof plate of the anterior neural tube, the isthmus and the midbrain floor plate. Subsequent studies demonstrated that RMST was closely related to neuronal differentiations 27. Studies also have shown that together with sex determining region Y‐box 2 (SOX2), RMST could co‐regulate many downstream genes involved in neurogenesis. In Ng *et al*.'s RNA interference and genome‐wide SOX2‐binding studies, RMST was found to be indispensable for SOX2's combination with promoter regions of neurogenic transcription factors 27.

Yang *et al*. sequenced eight paired non‐cancer samples and TNBC samples, and identified several abnormally expressed lncRNAs, among which, compared to adjacent non‐cancer breast tissues, RMST was significantly down‐regulated in TNBC 28. Moreover, low RMST were also associated with poor outcomes and worse prognosis than higher RMST, suggesting the cancer‐suppression roles of RMST in breast cancer 28. However, Yang's study had some limitations: (*i*) they did not perform *in vivo* experiments or ectopic expressions, which could help with confirming core lncRNAs’ roles in TNBCs; (*ii*) they only conducted the strand‐specific and Poly‐A‐dependent RNA sequencing, which was likely to lead to the loss of lncRNAs without Poly‐A.

## Small nucleolar RNA host gene 12 (SNHG12)

Small nucleolar RNA host genes (SNHGs) have been reported to contribute to the progression of cancers. SNHG12 (also known as GAS5) is a novel lncRNA identified to be up‐regulated in several cancer cells, such as human osteosarcoma cell, nasopharyngeal carcinoma cell and human endometrial carcinoma 29, 30. The original identification of SNHG12 from a subtraction cDNA library depended on its increased abundance in growth‐arrested mouse NIH 3T3 fibroblasts 31. Subsequently, it has been shown that it was the alterations of the biodegradation rate rather than the transcription rate that regulated the expression levels of SNHG12 32. Mourtada *et al*. found that overexpression of certain SNHG12 transcripts induces growth arrest and apoptosis in human breast cancer cell lines. SNHG12 levels were down‐regulated significantly in human breast cancer cell lines, suggesting that the decrease in SNHG12 expressions might play important roles in the oncogenesis. According to their study, in the breast cancer cell lines, SNHG12 expressions were generally inversely correlated to tumorigenic behaviours 11.

SNHG12 played important roles in cancer cell proliferation and migration. Wang *et al*. discovered that SNHG12 was up‐regulated in colorectal cancer tissues and cells. They also detected the effect of SNHG12 on cell proliferation, cell cycle, apoptosis and the related proteins expression in CRC cells 33. Subsequently, Wang *et al*. utilized RNA sequencing (RNA‐seq) to explore the lncRNAs expression profiles in TNBC and identified that SNHG12 was remarkably increased in TNBC 34. Subsequently, they determined that SNHG12 is significantly up‐regulated in 102 TNBC tumour tissues compared to 95 non‐cancerous breast tissues by qRT‐PCR (*P* < 0.001). The expression levels of SNHG12 were statistically related to the lymph node metastasis (*P* = 0.041) and the tumour size (*P* = 0.012) 34. Patients with higher SNHG12 expression levels were inclined to have larger tumours and metastatic lymph nodes. Mechanistic investigations show that SNHG12 is a direct transcriptional target of c‐MYC. The depletion of c‐MYC by siRNA in TNBC cell lines BT‐549 and MDA‐MB‐231 significantly reduced SNHG12 transcript levels (*P* < 0.05) 34. In addition, SNHG12 levels were markedly increased in BT‐549 and MDA‐MB‐231 cells transfected with c‐MYC overexpression plasmid. Silencing SNHG12 expression inhibits TNBC cells proliferation and apoptosis promotion, whereas enforced expression of SNHG12 promoted TNBC cells proliferation and migration. In addition, they reveal that SNHG12 was mainly located in cytoplasm and may regulate MMP13 expression to promote cells migration 34. However, SNHG12's biological roles still have not yet been identified in *in vivo* experiments, and the mechanisms by which SNHG12 mediates the apoptosis or proliferation remains unclear.

## SnaR

SnaR, a double‐stranded lncRNA of 117 nt, is transcribed by RNA polymerase‐III linked with nuclear factor 90 (NF90). SnaR binds important proteins implicated in multiple cellular functions by *in vivo* cross‐linking followed by immune‐precipitation, indicating the possibility that it has critical roles in the regulation of cancer's initiation and progression 35. Several snaR transcripts have been found to be associated with ribosomes in cytoplasm 36. What is more, it has been observed that these regulatory roles in cell growth and gene translation are species specific and tissue specific. Lee H *et al*. reported that in 5‐FU‐resistant colon cancer cells, snaR was down‐regulated. Additionally, after 5‐FU treatment, the down‐regulation could decrease Annexin V‐positive (ANN+) apoptotic cells, indicating that snaR might negatively regulate cell growth and tissue development after 5‐FU treatment 37. In Lee *et al*.'s study, diverse breast cancer cell lines based on molecular subtype, namely BT20, BT474, T47D, SKBR3, MCF7 and MDA‐MB‐231, were used to explore the role of lncRNAs 38. Although various lncRNAs were expressed highly in each cell line, snaR and ANRIL were identified as being predominantly up‐regulated in MDA‐MB‐231 cell line and the hormone receptor‐expressing cell line (MCF7). Particularly, snaR was shown to be 16.82 ± 3.44‐fold more highly expressed in the TNBC cells than control 38. The invasion, migration and proliferation of TNBC cells could be significantly inhibited after snaR‐knockdown. So, if the knockdown of snaR can be applied clinically to TNBC, it would provide an innovative treatment for such cancer.

## Conclusion

TNBC generally behaves more aggressively with a relatively poorer prognosis than other phenotypes of breast cancer. Our understanding of the cellular origin and pathogenic mechanisms of TNBC remains fragmented. In the past several years, the researchers have witnessed a steep rise of interest in the study of lncRNAs in many diseases, including various kinds of cancer. Recent studies have identified a series of dysregulated lncRNAs in TNBC. These lncRNAs may serve as biomarkers and therapeutic targets for TNBC in the future.

## Conflict of interest

We have no conflicts of interests.
